# Outcomes of Cataract Surgery in Eyes With Diabetic Retinopathy at a Tertiary Care Hospital in Malaysia

**DOI:** 10.7759/cureus.91437

**Published:** 2025-09-01

**Authors:** Faradatul Aisyah Abdul Aziz, Nor Anita Omar, Nor Idahriani Muhd Nor, Jemaima Che Hamzah

**Affiliations:** 1 Ophthalmology, Hospital Sultanah Nur Zahirah, Kuala Terengganu, MYS; 2 Ophthalmology, Hospital Kuala Lumpur, Kuala Lumpur, MYS; 3 Ophthalmology, Faculty of Medicine, Universiti Kebangsaan Malaysia, Kuala Lumpur, MYS

**Keywords:** cataract, cataract surgery, diabetic retinopathy, eyes, outcome, postoperative

## Abstract

Purpose: The study aimed to assess the clinical outcomes of cataract surgery in eyes with diabetic retinopathy (DR).

Methods: This was a retrospective study of 150 eyes (135 patients) with significant cataracts and different stages of DR, with or without maculopathy, who underwent cataract surgery from January 2020 until September 2022 at Hospital Sultanah Nur Zahirah (HSNZ), Kuala Terengganu, Malaysia. All patients had a minimum of six weeks of postoperative follow-up. The demographic data, stages of DR, and clinical data of the eyes were recorded preoperatively and during the six-week postoperative follow-up. Intraoperative and postoperative complications were recorded and analyzed.

Results: A total of 135 patients (150 eyes) with a mean age of 64 ± 9.34 were enrolled. The patients were primarily Malay (131 patients, 97.3%) and Chinese (four patients, 2.7%). Stages of DR were divided into five categories: non-proliferative diabetic retinopathy (NPDR) without maculopathy (73 eyes, 48.7%), NPDR with maculopathy (36 eyes, 24%), proliferative diabetic retinopathy (PDR) without maculopathy (16 eyes, 10.6%), PDR with maculopathy (16 eyes, 10.6%), and advanced diabetic eye disease (ADED) (nine eyes, 6%). The mean preoperative visual acuity (VA) was 6/60. Postoperative best corrected visual acuity (BCVA) varied according to the stages of DR. NPDR without maculopathy showed good visual improvement with a mean of 6/9. For NPDR with maculopathy, the mean was 6/12; for PDR without maculopathy, it was 6/12; and for PDR with maculopathy, it was 6/24. For ADED, the postoperative mean was the poorest compared to other groups at 6/48.

Conclusion: The outcomes of cataract surgery in the eyes of patients with DR depend on the severity of the disease. Early detection and appropriate management of DR are crucial for better postoperative VA.

## Introduction

One of the main consequences of diabetes mellitus (DM) and the primary cause of visual loss in the working-age group is diabetic retinopathy (DR) [1]. It has long been understood that DR is a microvascular condition. Hyperglycemia is a crucial factor in the pathophysiology of retinal microvascular injury. The polyol pathway, the buildup of advanced glycation end products (AGEs), the protein kinase C (PKC) pathway, and the hexosamine pathway are the metabolic processes linked to hyperglycemia-induced vascular injury [2].

Clinical signs of vascular anomalies in the retina are used to diagnose DR. There are two clinical stages of DR: non-proliferative diabetic retinopathy (NPDR) and proliferative diabetic retinopathy (PDR) [3]. Increased vascular permeability and capillary occlusion are two main clinical signs in the retinal vasculature that occur in NPDR, which is the first stage of DR. Retinal diseases such as microaneurysms, intraretinal hemorrhages, cotton wool spots, intraretinal microvascular abnormalities (IRMA), and hard exudates can be identified clinically and through fundus photography at this stage, even though patients may not exhibit any symptoms.

Neovascularization is a hallmark of PDR, a more severe form of DR. Once the newly formed abnormal arteries enter the vitreous, patients may experience severe vision impairment. This can lead to either pre-retinal hemorrhage or vitreous hemorrhage, which may cause tractional retinal detachment (TRD) with or without rhegmatogenous retinal detachment (RRD). Diabetic macular edema is the most frequent cause of visual loss in DR patients.

Compared to individuals without diabetes, diabetics experience cataract development 10 years earlier. Diabetic patients who undergo cataract surgery are more likely to face postoperative management problems [4]. Dowler et al. demonstrated that the two main determinants of postoperative visual acuity (VA) are the degree of retinopathy and the presence of preoperative maculopathy in a meta-analysis of 10 studies related to the visual outcome of retinopathy [5]. According to Becker et al., individuals with diabetes who receive an earlier diagnosis are more prone to developing cataracts [6].

The management of retinopathy can be hindered by opaque media, making it difficult to adequately monitor and manage DR through laser photocoagulation. In this study, the surgical outcomes are analyzed based on their impact on visual improvement and the progression of retinopathy [5]. The findings highlight the elevated risk of visual loss following surgery and the importance of early DR control. As phacoemulsification has become more widely accessible, the study also investigates how different surgical techniques relate to the progression of retinopathy.

Prior research has shown that posterior capsular rupture in DR has an odds ratio (OR) of 1.74 (1.34-2.27), which raises the risk of endophthalmitis, retinal detachment, and postoperative cystoid macular edema [7-9]. Worldwide, there are not many studies examining intraoperative challenges and problems in DR patients. According to one study, patients with other ocular pathologies exhibited less improvement in self-assessed visual function than those without ocular comorbidities, such as DR [10].

The objective of this study was to determine and compare the outcomes of cataract surgery in eyes with DR, including postoperative VA and intraoperative and postoperative complications based on the stages of DR.

## Materials and methods

The records of every diabetic patient who had cataract surgery at Hospital Sultanah Nur Zahirah (HSNZ), Kuala Terengganu, Malaysia, during the 33-month period from January 2020 to September 2022, were retrospectively analyzed. The National Eye Database (NED), Cataract Surgical Registry (CSR), and Electronic Medical Record (EMR) at HSNZ were the sources of all the entries. A total of 150 eyes were operated on, involving 135 consecutive patients. Based on fundus findings, the existence and type of preoperative retinopathy were determined. Stages of DR were classified into five categories: NPDR without maculopathy, NPDR with maculopathy, PDR without maculopathy, PDR with maculopathy, and advanced diabetic eye disease (ADED).

Preoperative treatments for retinopathy were recorded, such as pan-retinal photocoagulation (PRP) or focal/grid laser treatment, intravitreal anti-VEGF injections, and vitrectomy. Preoperative best-corrected Snellen VA was measured, and preoperative treatment was generally conducted in accordance with the standard Diabetic Retinopathy Study (DRS) and Early Treatment Diabetic Retinopathy Study (ETDRS) recommendations [11,12]. Either extracapsular extraction or phacoemulsification was performed. Combined procedures involving corneal grafting or trabeculectomy were excluded, but vitrectomized eyes were included in this study. All patients had a minimum of six weeks of postoperative follow-up. The demographic data, stages of DR, and clinical data of the eyes were recorded both preoperatively and at the six-week postoperative follow-up. Intraoperative and postoperative complications were documented and analyzed. The best VA attained at least six weeks after surgery was reported as postoperative VA.

Inclusion criteria were that patients underwent cataract surgery at HSNZ over a 33-month period from January 2020 to September 2022, with complete data on preoperative best-corrected visual acuity (BCVA), as reported in the CSR, and available data on postoperative BCVA at least six weeks postoperatively.

Ocular comorbidities other than DR, such as glaucoma, age-related macular degeneration (AMD), Fuchs' endothelial dystrophy, pseudoexfoliation, or other eye diseases, as well as combined surgeries and missing data on refractive error or preoperative/postoperative BCVA, were excluded.

A good surgical outcome is defined as postoperative VA of 6/12 or better, with fewer intraoperative and postoperative complications, comparable to those in other studies.

## Results

In the study, 135 patients were included, comprising 52 male and 83 female patients. A total of 150 eyes underwent surgery, with 75 cases performed on the right eye and 75 cases on the left eye. The average age of the participants was 64.0 ± 9.34 years. The mean duration of diabetes from the time of diagnosis was 11.7 years. All participants had type 2 diabetes, and 60% were receiving insulin treatment. Systemic hypertension was observed in 26 patients (19%) out of the total. The average HbA1c levels were 9.3 ± 1.7%, and the average duration of diabetes was 16.0 ± 11.0 years. Most patients (48.7%) had NPDR without maculopathy. The rest of the patients had NPDR with maculopathy (36 eyes, 24%), PDR without maculopathy (16 eyes, 10.6%), PDR with maculopathy (16 eyes, 10.6%), and ADED (nine eyes, 6%).

In 135 cases, the cataractous lens was phacoemulsified through a corneal incision. A posterior chamber intraocular lens (IOL) was implanted in all cases. Thirteen cases underwent extracapsular extraction, primarily due to hard cataracts that could not be managed with phacoemulsification. The remaining two cases underwent intracapsular cataract extraction because of significant zonular dialysis with phacodonesis. Table 1 shows the demographic data of the patients.

**Table 1:** Demographic data and clinical characteristic of patients PDR = Proliferative diabetic retinopathy; NPDR = Non-proliferative diabetic retinopathy; ADED = Advanced diabetic eye disease; ECCE = Extracapsular cataract extraction; ICCE = Intracapsular cataract extraction; DR = Diabetic retinopathy

Variable	Value (n)	Frequency (%)
Total eyes	150	
Mean age (SD)	64.0 ± 9.34	
Gender		
Male	52	38.5
Female	83	61.5
Laterality		
Right eye	75	50
Left eye	75	50
Race		
Malay	131	97.3
Chinese	4	2.7
Stages of DR		
NPDR	73	48.7
NPDR with maculopathy	36	24
PDR	16	10.6
PDR with maculopathy	16	10.6
ADED	9	6
Type of cataract surgery		
Phacoemulsification	135	90
ECCE	13	8.7
ICCE	2	1.3

Table 2 displays preoperative VA in every group based on DR status. Table 3 presents postoperative VA at six weeks. In patients with NPDR without maculopathy, 76% (56 eyes) achieved postoperative BCVA better than 6/12. For patients with NPDR with maculopathy and PDR, at least more than 50% were able to obtain vision of at least 6/18 or better: 76% in NPDR with maculopathy and 52% in PDR. Only one ADED patient was able to achieve vision better than 6/12, as shown in Table 3.

**Table 2:** Preoperative visual acuity by diabetic retinopathy status PDR = Proliferative diabetic retinopathy; NPDR = Non-proliferative diabetic retinopathy; ADED = Advanced diabetic eye disease

Visual acuity	NPDR (n)	NPDR with maculopathy (n)	PDR (n)	PDR with maculopathy (n)	ADED (n)
						<6/12	0	0	0	0	0
6/12-6/18	10	4	2	2	1
6/24-6/60	29	14	7	4	1
>6/60-3/60	20	6	1	3	1
>3/60	14	12	6	7	6

**Table 3:** Postoperative visual acuity by diabetic retinopathy status PDR = Proliferative diabetic retinopathy; NPDR = Non-proliferative diabetic retinopathy; ADED = Advanced diabetic eye disease

Visual acuity	NPDR (n)	NPDR with maculopathy (n)	PDR (n)	PDR with maculopathy (n)	ADED (n)
						<6/12	56	14	6	2	1
6/12-6/18	10	13	3	3	1
6/24-6/60	4	7	4	9	2
>6/60-3/60	2	2	1	1	1
>3/60	1	0	2	1	4

Following cataract surgery, none of the patients experienced anterior segment complications, such as an increase in intraocular pressure, fibrin or exudation, posterior synechia development, pupillary obstruction, or neovascularization of the iris. About 30% were diagnosed with macular edema in the first operative year. Patients with maculopathy were treated with a topical anti-inflammatory drug (nepafenac) and did not require intravitreal anti-VEGF injections. Patients in the PDR group also did not receive any laser intervention before the surgery. After treatment, some patients were able to maintain their VA, and others gained better vision compared to before cataract surgery. None of the patients developed ischemic macular edema. The incidence of macular edema did not change statistically significantly between eyes that had surgery and those that did not (P=0.11). During the first year after surgery, no patient required yttrium-aluminum-garnet (YAG) laser capsulotomy. 

Intraoperatively, 89.3% underwent cataract surgery without complications. Complications encountered during this study included posterior capsular rupture, zonular dialysis, descemet tear, and bleeding from the iris. The results are shown in Table 4.

**Table 4:** Intraoperative and six-week postoperative complications

Complication	Total eyes (n, %)
		Intraoperative complication	
None	134 (89.3)
Posterior capsular rupture	8 (5)
Zonular dialysis	4 (3)
Descemet tear	3 (2)
Bleeding from the iris	1 (0.7)
Postoperative complication	
None	98 (65)
Macula edema	43 (30)
Macula scar	2 (1)
Other	6 (4)

## Discussion

In this study, phacoemulsification and posterior chamber IOL implantation improved VA in patients with cataract and NPDR without maculopathy. Overall, 79 eyes showed visual improvement, achieving a VA of 6/12. In patients with NPDR without maculopathy, 76% (56 eyes) achieved postoperative BCVA better than 6/12. For patients with NPDR with maculopathy and PDR, at least more than 50% were able to obtain vision of at least 6/18 or better: 76% in NPDR with maculopathy and 52% in PDR. Only one ADED patient was able to achieve vision better than 6/12. Following cataract surgery, none of the patients experienced anterior segment complications, such as an increase in intraocular pressure, fibrin or exudation, posterior synechia development, pupillary obstruction, or neovascularization of the iris. About 30% were diagnosed with macular edema in the first operative year. Our study's visual outcome results are similar to those of other research projects utilizing contemporary surgical methods [13-15].

Complications of cataract surgery in diabetic patients have also been documented in numerous studies. According to Cunliffe et al., diabetic patients had a higher incidence of macular edema and severe ocular inflammation after extracapsular cataract excision. Neovascular glaucoma and postoperative rubeosis were also observed [15]. Patients with quiescent proliferative retinopathy or non-proliferative retinopathy are more likely to develop posterior capsule opacification. However, in the first year postoperatively, none of our subjects required YAG laser capsulotomy.

We found that the intraoperative complication rate in our study was 10.7%, which is quite high. The most common complication was posterior capsular rupture (5%). Chancellor et al. showed that the greater incidence of progressive cataracts and poor pupil dilatation intraoperatively makes phacoemulsification in diabetics more difficult. In their large-scale research, they found that diabetic patients had roughly 1.6 times higher risks of posterior capsule rupture [16]. Patients with diabetes exhibited a greater frequency of posterior capsule rupture in their eyes compared to those without the condition (2.1% vs. 1.6%; P<0.0001). Furthermore, they discovered that diabetes (OR 1.263) was a risk factor for posterior capsule rupture, regardless of whether retinopathy was present [16]. In a short retrospective analysis of 150 diabetic patients' eyes, Mittra et al. reported a posterior capsule rupture rate of 12% among all surgeons and 4% among senior surgeons [17].

The results of our research align with these findings. Compared to patients with DR without maculopathy, those with pre-existing maculopathy had poorer visual outcomes [16,17]. The development of persistent, sight-threatening macular edema seems to be managed by preoperative laser treatment. Postoperative care, on the other hand, may not be as successful. When compared to patients treated postoperatively or not at all, the preoperatively treated group with DR without macular edema showed better visual outcomes and less retinopathy progression. This presents a challenge, especially for patients with thick cataracts requiring subsequent maculopathy therapy [4-6].

According to our study, NPDR cases seem to have better visual outcomes and control over retinopathy than PDR cases, regardless of the presence of maculopathy. This is consistent with Dowler et al.'s meta-analysis, which demonstrated that poor VA is always linked to the existence of active, untreated PDR. The likelihood of postoperative VA worse than 6/12 is three times higher for treated quiescent PDR. Compared to patients without maculopathy, those with maculopathy before surgery are six times more likely to have postoperative vision worse than 6/12. This can also be attributed to clear instructions for preoperative laser treatment and earlier detection of proliferative changes [5]. Additionally, patients with PDR may only undergo cataract surgery if their retinopathy has been properly treated, which could indicate a selection bias. According to Hykin et al.'s study, there were no cases of acute fibrinous anterior uveitis or neovascularization of the iris following cataract surgery [18].

Many previous studies have identified several markers for high-risk diabetic patients, including age over 64, female gender, poor glycemic control, insulin dependence, and, most notably, the degree of pre-existing retinopathy [18-21]. These factors should be considered during preoperative planning and patient counseling, assisting physicians in determining whether cataract surgery is necessary and when. Given that postoperative laser treatment is likely to be needed in high-risk patients, it would be prudent to use a large optic IOL and perform a larger capsulorhexis during surgery to improve photocoagulation of the peripheral retina [15].

One limitation of this retrospective study was the challenging and insufficient data collection. Even though 33 months were spent recruiting patients, especially for the PDR and ADED groups, there weren't many cases. Significant variations in visual results and retinopathy progression would require a larger prospective series with more precise selection criteria and retinopathy grading.

The outcome of cataract surgery in eyes with DR depends on the severity of the disease. Early detection and appropriate management can lead to better postoperative VA. Following uncomplicated cataract surgery with posterior chamber lens implantation, patients with early DR had a good prognosis. In this patient population, the progression of DR appears to be unaffected by modern cataract surgery. However, if diabetic macular edema develops, these patients should be advised that the prognosis for their vision may be uncertain. Careful monitoring for the early identification and management of diabetic macular edema is essential to maintain the visual improvement achieved through cataract surgery.

## Conclusions

In conclusion, this study described and measured the percentage of intraoperative complications and visual results in the eyes of patients with DR following cataract surgery. In the early stages of DR, we found that cataract surgery significantly improved postoperative VA. At six weeks after surgery, the VA of three Snellen lines or more was improved in 79 eyes of diabetic patients. On the other hand, diabetes was negatively associated with final VA, and eyes with higher ETDRS scores had lower postoperative VA.
